# How to Break the Cycle of Low Workforce Diversity: A Model for Change

**DOI:** 10.1371/journal.pone.0133208

**Published:** 2015-07-28

**Authors:** Katherine R. O’Brien, Marten Scheffer, Egbert H. van Nes, Romy van der Lee

**Affiliations:** 1 School of Chemical Engineering, The University of Queensland, St Lucia, 4072, Queensland, Australia; 2 Aquatic Ecology and Water Quality Management, Wageningen University, 6700 AA, Wageningen, The Netherlands; 3 Social and Organizational Psychology Unit, Institute of Psychology, Leiden University, P.O. Box 9555, 2300 RB, Leiden, The Netherlands; University of Maribor, SLOVENIA

## Abstract

Social justice concerns but also perceived business advantage are behind a widespread drive to increase workplace diversity. However, dominance in terms of ethnicity, gender or other aspects of diversity has been resistant to change in many sectors. The different factors which contribute to low diversity are often hotly contested and difficult to untangle. We propose that many of the barriers to change arise from self-reinforcing feedbacks between low group diversity and inclusivity. Using a dynamic model, we demonstrate how bias in employee appointment and departure can trap organizations in a state with much lower diversity than the applicant pool: a workforce diversity “poverty trap”. Our results also illustrate that if turnover rate is low, employee diversity takes a very long time to change, even in the absence of any bias. The predicted rate of change in workforce composition depends on the rate at which employees enter and leave the organization, and on three measures of inclusion: applicant diversity, appointment bias and departure bias. Quantifying these three inclusion measures is the basis of a new, practical framework to identify barriers and opportunities to increasing workforce diversity. Because we used a systems approach to investigate underlying feedback mechanisms rather than context-specific causes of low workforce diversity, our results are applicable across a wide range of settings.

## Introduction

Numerous companies, professions, government agencies and leadership teams worldwide are actively working to increase workforce diversity [1]. Incentives to increase diversity include access to larger talent pool, improvements in team creativity, innovation and problem-solving, return on investment in training and greater connection to clients and customers: this is the “business case” for diversity [2–5]. There are also important social and ethical drivers for increasing diversity, such as overcoming disadvantage in access to education, health and economic resources, which can benefit both individuals and communities: diversity as “the right thing to do” [4,6,7].

Workforce diversity has increased in many sectors over recent decades, but change is sometimes slow, or confined to only one area. For example, African-Americans are well-represented in police departments in large US cities, but account for only 5–6% of police in smaller towns [8]. Women account for approximately half of all science and medicine graduates in some countries [9,10], but remain poorly represented in leadership roles and in traditionally male fields, such as mathematics and surgery [11–13], and males from European or English-speaking countries dominate scientific publications globally [14,15]. Gender diversity in the medical profession has increased, but indigenous people and other ethnic groups are under-represented [11,16,17].

Where workforce diversity has been resistant to change, there are often many factors involved, making it difficult to determine which key barrier or barriers to prioritize. For example, workplace culture [12,18], socio-cultural factors [19], implicit bias [20,21], career preferences [22], family responsibilities [23], innate gender differences [24]- for a discussion see [25], lack of role models [26], stereotype threat [27], training opportunities [28], and differences in resource allocation and service duties [29] are all proposed to play a role in low female retention and seniority in Science, Technology, Engineering and Mathematics (STEM) professions. Similar mechanisms are blamed for the observed horizontal and vertical gender stratification in medicine [10] and in leadership positions more generally [30].

It is difficult to identify a single barrier to changing workforce diversity because of the inherent complexity of group dynamics. Diversity has the potential to increase group performance (particularly creativity and innovation) by expanding the perspectives and knowledge available to the group, according to information/decision making theories [5,31,32]. Social categorization and similarity-attraction theories, however, suggest that diversity can undermine group performance through reducing cohesion, trust and communication and increasing intergroup bias and conflict [31–33]. Furthermore, different classes and dimensions of diversity (e.g. values, education, personality and demographic characteristics such gender or race) affect group dynamics differently [34,35] and can create “faultlines” which undermine group processes [36–38].

**Fig 1:**
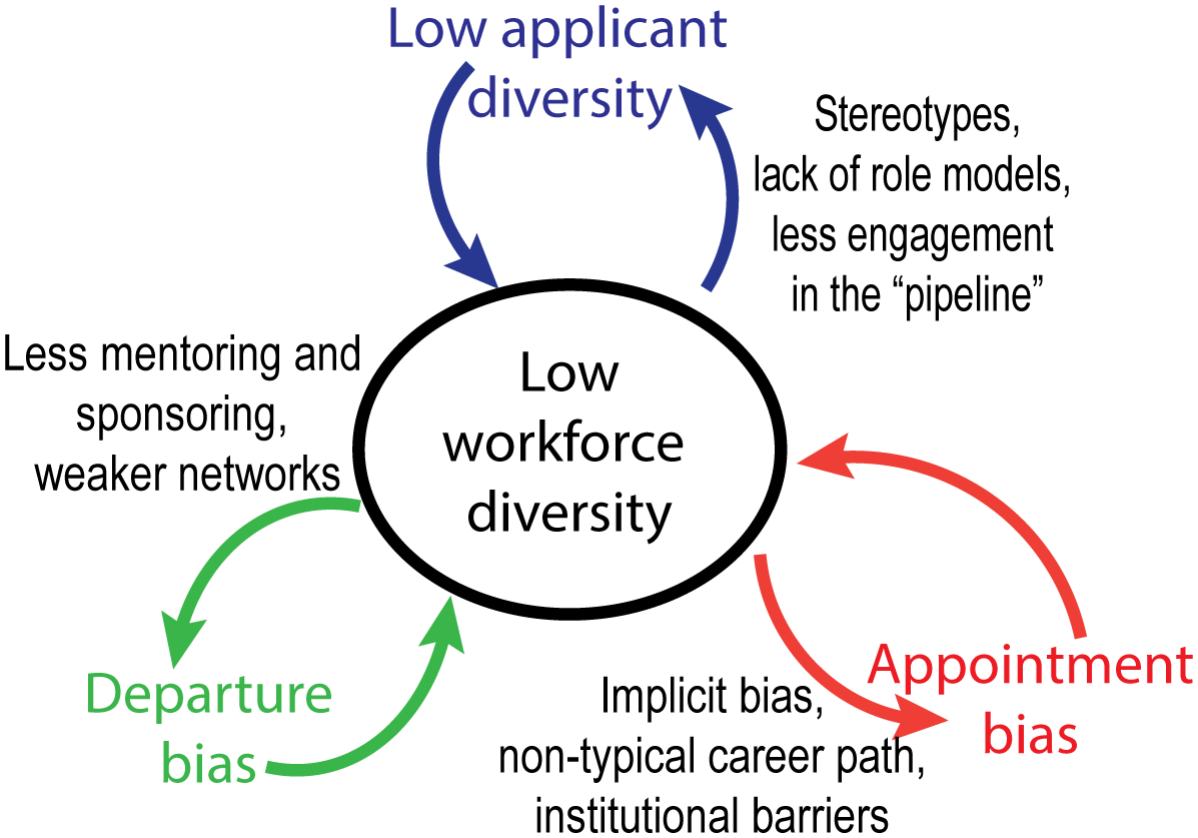
Low diversity can be reinforced by feedbacks with three measures of inclusion: applicant diversity, appointment bias and departure bias.

This paper uses mathematical modelling to investigate long-term changes in workforce composition. To deal with the inherent complexity of diverse groups, we use a system dynamics approach. Our model investigates underlying feedback mechanisms rather than context-specific causal factors, because feedbacks are often important levers for change in complex systems (Meadows 2008). This systems approach means that our results are not restricted to a specific profession, organization or point in time, but will be applicable across a broad range of settings. Specifically, we model two self-reinforcing feedbacks between diversity and inclusivity which have the potential to perpetuate group homogeneity (Fig 1). From the model, we define the key parameters which control the rate at which workforce composition can change, and demonstrate how this information can be used to identify traps and opportunities for changing workforce diversity.

## Methods

We developed a dynamic model of workforce composition from a balance of employees appointed to and departing from an organisation. The model predicts how appointment and departure bias affect workforce demographics over time, when diversity is higher in the applicant pool than in the workforce. For simplicity, we considered only one, easily measurable dimension of diversity (e.g. gender or ethnicity), with only two groups of possible employees (a dominant group, to which the majority of employees belong, and a non-dominant group which accounts for all other employees). Therefore we did not need to calculate the diversity index based on group composition [35], and so we use a simpler diversity metric: the proportion of the organization from the non-dominant group.

The model is described using the terms “organization” and “employees”, but can readily be applied to other examples, such as a shift in the diversity of a professional group (e.g. health, law enforcement or education) to better reflect the socio-economic, racial, gender and ethnic diversity of the community which they serve.

The rate of change in the total number of employees (*T*_*Employee*_) depends on the rates at which employees are appointed to (*α*_*Appoint*_, people/y) and depart from (*α*_*Depart*_, people/y) the organisation. Assuming a large organisation, we treat *T*_*Employee*_ as a continuous variable and accordingly define the rate of change in number of employees d*T*_*Employee*_/*dt* as follows:

$$\frac{dT_{Employee}}{dt}=\alpha_{Appoint}-\alpha_{Depart}$$ (1)

All employees belong to either the dominant or non-dominant group, hence the number of employees *T*_*Employee*_ is the sum of the number of employees from the dominant group *D*_*Employees*_ and from the non-dominant group *ND*_*Employees*_. The rate at which employees from the non-dominant group are appointed to the organisation will therefore depend on the overall appointment rate (*α*_*Appoint*_, people/y), the proportion of applicants from the non-dominant group (applicant diversity *d*_*Applicant*_ = *ND*_*Applicant*_ */T*_*Applicant*_,), and whether applicants from the non-dominant group are more or less likely than other applicants to be appointed, referred to hereafter as appointment bias (*Bias*_*Appoint*_, appointment diversity compared to applicant diversity). Similarly, the rate at which employees from the non-dominant group depart from the organisation will depend on the overall departure rate (*α*_*Depart*_, people/y), employee diversity (*x*_*Employee*_ =* ND*_*Employee*_ */T*_*Employee*_), and whether employees from the non-dominant group are more or less likely to leave than other employees (*Bias*_*Depart*_, diversity of departing employees compared to diversity of all employees). Therefore the rate of change in number of employees from the non-dominant group *ND*_*Employees*_ can be written:

$$\frac{dND_{Employee}}{dt}=\alpha_{Appoint}d_{Applcant}Bias_{Appoint}-\alpha_{Depart}x_{Employee}Bias_{Depart}$$ (2)

where appointment bias *Bias*_*Appoint*_, the ratio of appointee diversity compared to applicant diversity, can also be written as the appointment success rate of applicants from the non-dominant group compared to overall appointment success rate:

$$Bias_{Appoint}=\frac{d_{Appoint}}{d_{Applicant}}=\frac{\frac{ND_{Appoint}}{T_{Appoint}}}{\frac{ND_{Applicant}}{T_{Applicant}}}=\frac{\frac{ND_{Appoint}}{ND_{Applicant}}}{\frac{T_{Appoint}}{T_{Applicant}}}$$ (3)

Similarly, departure bias *Bias*_*Depart*_ is the diversity of those leaving the organization compared to overall employee diversity [4], and can also be written as the departure rate of employees from the non-dominant group compared to the overall employee departure rate:

$$Bias_{Depart}=\frac{d_{Depart}}{x_{Employee}}=\frac{\frac{ND_{Depart}}{T_{Depart}}}{\frac{ND_{Employee}}{T_{Employee}}}=\frac{\frac{ND_{Depart}}{ND_{Employee}}}{\frac{T_{Depart}}{T_{Employee}}}$$ (4)

To investigate the impact of non-linear feedbacks between applicant diversity and appointment and departure bias, we considered the simplest possible case, an organisation of fixed size (*dT*_*Employee*_ */dt* = 0). This can only occur when the rate of appointment matches the rate of departure (i.e. *α*_*Appoint*_ =* α*_*Depart*_ =* α*_*Turnover*_), which can be written as turnover rate *r*_*T*_ by dividing the rates by the total number of employees (*r*_*T*_ =* α*_*Turnover*_ */T*_*Employee*_). Normalizing Eq 2 by dividing throughout by the total number of employees *T*_*Employee*_, and rearranging in terms of employee diversity (*x*_*Employee*_ =* ND*_*Employee*_ */T*_*Employee*_) yields our final model for rate of change in employee diversity:

$$\frac{dx_{Employee}}{dt}=r_{T}\left(d_{Applicant}Bias_{Appoint}\left(x_{Employee}\right)-x_{Employee}Bias_{Depart}\left(x_{Employee}\right)\right)$$ (5)

assuming that both appointment and departure bias are affected by employee diversity. When there is no bias (i.e. *Bias*_*Appoint*_ = *Bias*_*Depart*_ = 1), Eq 5 can be solved analytically, and the time *t* taken to reach target diversity *x*_*Employee*_ *(t)* will depend on turnover rate *r*_*T*_, applicant diversity *d*_*A*_ and initial employee diversity *x*_*Employee*_ *(0)*:

$$t=\frac{1}{r_{T}}\text{ln}\left(\frac{d_{Applicant}-x_{Employee}\left(0\right)}{d_{Applicant}-x_{Employee}\left(t\right)}\right)$$ (6)

### Modelling Bias

We assume that both appointment and departure bias vary non-linearly with workforce diversity, and that bias is more likely to occur when employee diversity is very low. These assumptions are based on numerous studies documenting implicit bias against individuals who contradict common stereotypes or differ from the in-group [20,21,39,40], and observed issues with engagement, satisfaction, and mentoring which can increase turnover rate for people work in small minorities [35,41–43]. Appointment bias is non-linear because implicit bias can negatively affect how performance is assessed when people are “demographic misfits”, particularly when they contradict common stereotypes [20,41,43–45]. Similar non-linearity occurs in departure bias, because isolation can affect motivation, individual and group performance, networks, sponsorship and hence turnover [35,41,46,47]. Accumulation of small differentials in performance assessment can make a big difference in long-term career trajectory and management team composition [48], affecting the availability of role models, societal perceptions and the appeal of the organisation or profession to potential applicants, as summarized in Fig 1.

**Fig 2:**
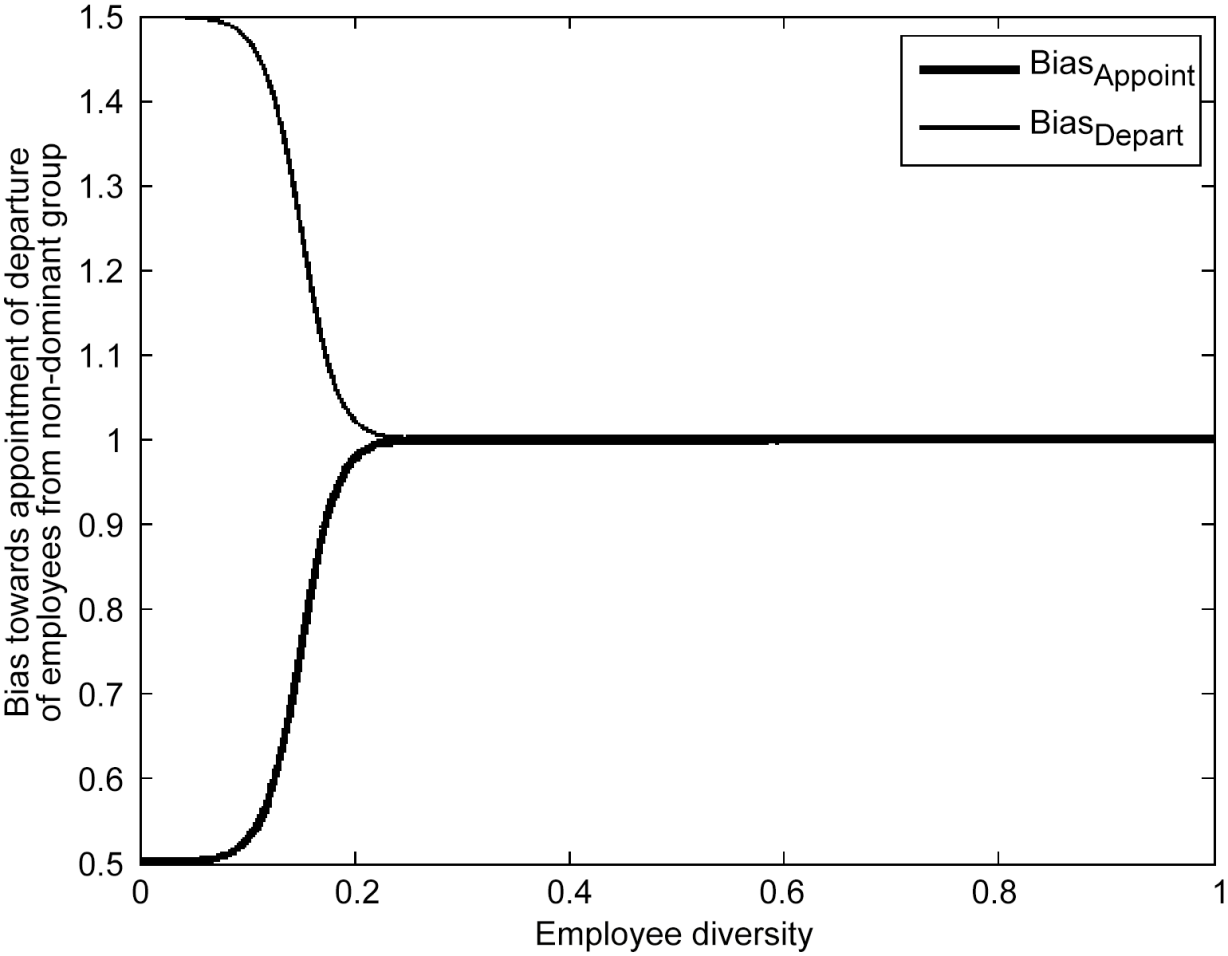
A mathematical model for appointment and departure bias (Eqs 7 and 8).

For mathematical representation of bias incorporating these two assumptions, we used the sigmoidal Hill function:

$$Bias_{Appoint}\left(x_{Employee}\right)=1-B_{Appoint}\frac{{\left(1-x_{Employee}\right)}^{m}}{{\left(1-x_{Employee}\right)}^{m}+h_{A}^{m}}$$ (7)

$$Bias_{Depart}\left(x_{Employee}\right)=1+B_{Depart}\frac{{\left(1-x_{Employee}\right)}^{n}}{{\left(1-x_{Employee}\right)}^{n}+h_{D}^{n}}$$ (8)

where the appointment and departure bias factors are assumed to be equal: *B*_*Appoint*_ =* B*_*Depart*_ = 0.5. This means that when employee diversity is very low (*x*_*Employee*_ << 1), individuals from the non-dominant group are up to 50% *less* likely to be appointed and up to 50% *more* likely to depart than others (Fig 2). The parameters *m* and *n* define how steeply appointment and departure bias, respectively, decline as the proportion of employees from the non-dominant group increases. If *m* and *n* are much larger than one, then appointment and departure bias are very strong for a homogeneous organization, and decline very rapidly as employee diversity approaches (1-*h*_*A*_) and (1-*h*_*D*_) respectively. We use the parameter values *h*_*A*_ = *h*_*D*_ = 0.85 and *m* = *n* = 50, so that bias was negligible (i.e. appointment and departure rates differed by less than 1%) once the non-dominant group accounted for more than 20% of the organization (Fig 2).

Parameters in Eqs 7 and 8 could not be quantified directly from data, but were chosen such that the bias models were broadly consistent with published literature, e.g. [21,35,46]. The parameter values and mathematical functions used in Eqs 7 and 8 were varied as follows in a sensitivity analysis of results: *B*_*Appoint*_, *B*_*Depart*_ = 0.25–0.75, *h*_*A*_, *h*_*D*_, = 0.75–0.95, m, n = 10–90. The effect of replacing the Hill functions of Eqs 7 and 8 with logistic or hyperbolic tangent functions was also assessed for model sensitivity.

### Model simulations

The model was run under four scenarios: *Scenario 1- No bias*: All individuals are equally likely to be appointed to or depart from the organization; *Scenario 2-Appointment bias*: Individuals from the non-dominant group are up to 50% less likely to be appointed than other applicants when employee diversity is very low (Fig 2); *Scenario 3-Departure Bias*: Individuals from the non-dominant group are up to 50% more likely to leave the organization than other employees when employee diversity is very low (Fig 2); *Scenario 4-Appointment and departure bias*: Individuals from the non-dominant group are subject to both appointment and departure bias when employee diversity is very low.

By definition, diversity in this two group system is maximum when half of the individuals are from each group, i.e. *x*_*Employee*_ = 0.5. Therefore the model was run for employee diversity and applicant diversity ranging from 0 to 0.5 under the four scenarios outlined above. Employee diversity was calculated over 80 years by discretizing Eq 5 with a time-step of one year. Equilibrium values of employee diversity were found by solving numerically for the values of *x*_*Employee*_ at which *dx*_*Employee*_ */dt* = 0 in Eq 5, using “Grind” for MATLAB (see http://www.dow.wau.nl/aew/grind/) (command “null”). The equilibrium values of employee diversity *x*_*Employee*_ calculated from Eq 5 ranged from 0 to 0.5 for applicant diversity *d*_*Applicant*_ between 0 and 0.5: hence for applicant diversity and initial employee diversity in the range 0–0.5, Eq 5 predicts *x*_*Employee*_ in the range 0 to 0.5. This meant that we were able to model employee diversity in the non-dominant group without needing to model employee diversity in the dominant group simultaneously.

## Results

**Fig 3:**
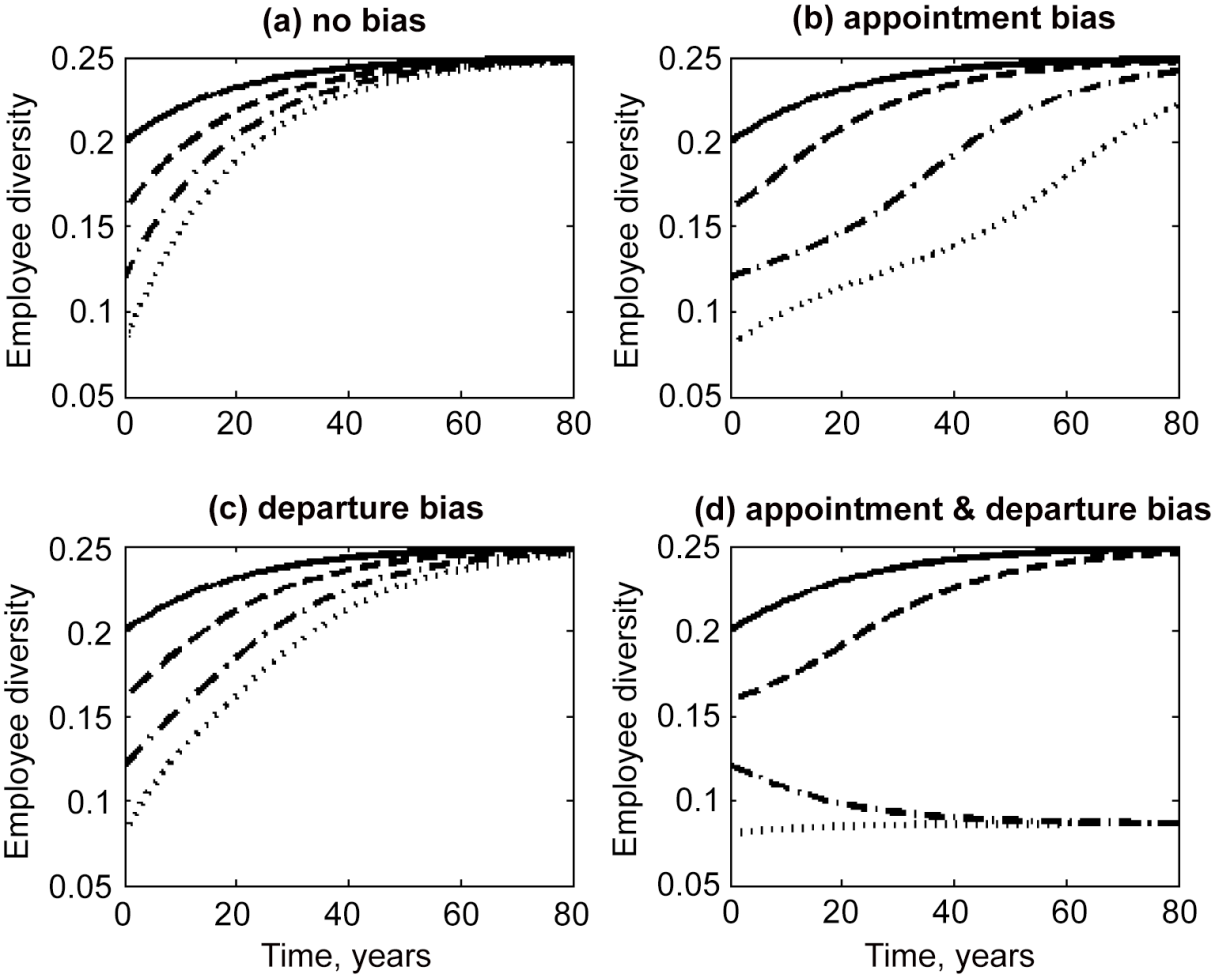
Change in employee diversity over time predicted from Eq 5, for turnover rate of 0.05 per year, applicant diversity of 0.25 and initial employee diversity of 0.08, 0.12, 0.16 and 0.20.

**Fig 4:**
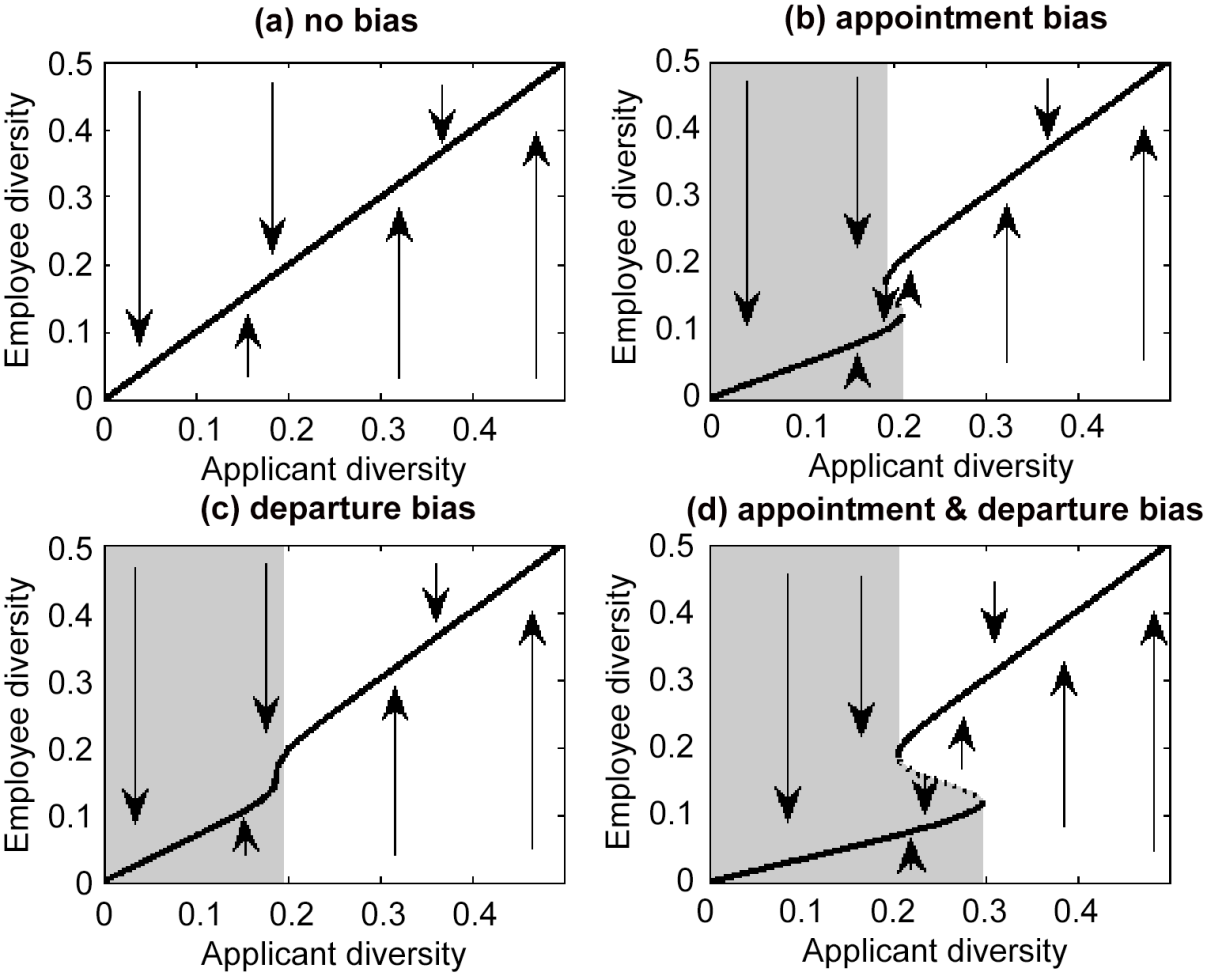
Predicted relationship between applicant diversity and equilibrium employee diversity: solid line and dashed lines represent stable and unstable equilibriums respectively. In the shaded area, the equilibrium employee diversity is less than the applicant diversity: the organization is trapped in a low diversity state due to bias in appointment and/or departure. Arrows indicate trajectory.

When initial employee diversity is high enough, our model predicts that workforce composition will slowly approach that of the applicant pool. For applicant diversity of 0.25 (i.e. 25% of applicants from the non-dominant group), employee diversity was predicted to approach 0.25 for all scenarios when initial diversity was equal to 0.2 (Figs 3 and 4). However, it takes a very long time for workforce composition to change when annual employee turnover rate is 0.05 per year, which was the value assumed in our model. In absence of any bias, it will take almost 52 years for employee diversity to double from 0.12 to 0.24, and 26 years if turnover rate is increased to 0.1 (Eq 6). Clearly few organisations will remain constant in size over these long time periods, as assumed in this model, but that does not detract from the key results: turnover rate determines how quickly workforce composition can change, and for typical turnover rates it can take many decades for diversity to increase, even in absence of bias.

When initial employee diversity is low (< 0.2), the rate of change in workforce composition will be substantially slowed by appointment bias (Fig 3b), but departure bias alone had a negligible effect on the predicted time for employee diversity to reach applicant diversity (Fig 3c). Appointment bias has the largest impact on workforce composition in this model because it is multiplied by applicant diversity, which is higher than employee diversity (Eq 5).

According to our model, an organization can become stuck at low diversity if both appointment and departure are biased (Figs 3d and 4d). Under certain conditions, the non-linear relationships we used to describe feedbacks between workforce diversity and bias in appointment and departure generate three equilibrium points for the same applicant diversity: a stable equilibrium where employee diversity equals applicant diversity, a stable equilibrium at lower diversity, and an unstable equilibrium in between these two points.

For example, if applicant diversity is 0.25, our model predicts stable equilibrium at employee diversity 0.25 and 0.09, and an unstable equilibrium at 0.15. Thus if employee diversity is initially less than 0.09, diversity will increase initially, but stablize at 0.09, because individuals from the dominant group are more likely to be appointed and less likely to leave than others. Once the proportion of employees from the non-dominant group exceeds 0.15, employee diversity will increase to reach applicant diversity (0.25); however if it falls below 0.15, appointment and departure bias become strong enough to drive diversity down to 0.09, as shown in Figs 3 and 4.

Where more than one stable equilibrium point exists for employee diversity, there is therefore potential for hysteresis in workforce composition. To illustrate, an organisation may be “stuck” with much lower diversity than the applicant group, but a small increase in the number of employees from the non-dominant group could reduce appointment and departure bias, causing the trajectory of workforce composition to change so that diversity begins to rise rather than fall (e.g. shifting from 14% to 16% in Fig 3d). Conversely, a small decline in diversity could be very difficult to reverse if it shifts the workforce into a “diversity poverty trap”.

For the parameter values used in these simulations, hysteresis or “diversity poverty trap” was not observed for applicant diversity above 0.3 (Fig 4). For applicant diversity below 0.3, however, predicted employee diversity depends on whether appointment and/or departure bias were included. The numerical thresholds specified here are indicative only, and reflect the choice of parameters: they should not be used as quantitative targets. The real significance is that the model demonstrates how non-linear feedbacks between diversity and bias in appointment and departure can delay changes in workforce composition, perpetuate group homogeneity and thus undermine other efforts to increase diversity.

Our findings are robust across a range of parameter values and bias function formulations. Across all the formulations and parametrizations of Eqs 7 and 8 outlined in the Methods, equilibrium employee diversity was predicted to be less than applicant diversity when applicant diversity was low. The magnitude of hysteresis in the employee diversity-applicant diversity phase space (Fig 4) increases with bias function steepness (larger *m*, *n*) and magnitude of maximum bias (*B*_*Appoint*_, *B*_*Depart*_). Increasing the critical mass required to eliminate bias (~1-*h*) increases both the magnitude of the hysteresis, and the range of applicant diversity under which bias is predicted to suppress employee diversity. Replacing the Hill function in Eqs 7 and 8 with logistic and hyperbolic tangent functions produced similar results to those described above: where the bias functions were less steep, hysteresis was less severe etc. Under certain parameter combinations in the alternate bias function formulations (shallow bias function and/or low magnitude bias and/or low diversity threshold for ending bias), bifurcation did not occur, i.e. for all values of applicant diversity, only a single equilibrium value of employee diversity existed. Conversely, where the alternate bias functions were particularly steep and/or bias was large in magnitude and/or threshold for bias elimination was high, hysteresis was predicted for every bias scenario, even when departure bias occurred in absence of appointment bias.

## Discussion

Our model makes two important contributions to understanding the dynamics of workforce composition, addressing a significant research gap highlighted almost two decades ago by [31]. Firstly, the model predictions demonstrate how non-linear feedbacks between group homogeneity and inclusion can have long-term implications for workforce composition. Specifically, appointment and departure bias can substantially reduce the rate at workforce diversity can increase, and in some cases low diversity can become self-perpetuating. The stronger the magnitude or non-linearity of the bias and the higher the value of diversity required to eliminate the bias, the more likely that a diversity “poverty trap” will exist, reinforcing dominance of the organization by one group.

Secondly, our model provides a new and useful framework for addressing one of the biggest challenges to changing diversity: How to assess and prioritize the many interacting factors which affect workforce composition? Our systems approach has identified four key mechanisms which control long-term workforce diversity: the rate of appointment and departure (or employee turnover rate), and three measures of inclusion: applicant diversity, appointment bias and departure bias. Each of these factors can be quantified, where diversity data is available. Table 1 demonstrates how this information can be used in practical ways to change diversity, although the effectiveness of these actions in any organisation will depend on having a person or group with clear authority and accountability for diversity, as illustrated by a long-term large-scale study of US companies [49].

**Table 1:** Traps and opportunities for increasing workforce diversity.

Measures of inclusion	Traps: barriers to increasing diversity	Opportunities: information and actions which can be used to enhance diversity
Applicant diversity	Employee diversity is constrained by applicant diversity	- Report applicant diversity to track progress, and identify areas of opportunity or concern; - Design recruiting strategies to attract diverse applicants; - Identify and remove institutional barriers to applicant diversity (e.g. policies for part-time work and parental leave, flexible university entry); - “Pipeline” activities to increase the diversity of future talent pool.
Appointment bias (Eq 3)	If appointment bias < 1, applicants from under-represented groups are less likely to be appointed. Main causes are implicit bias, institutional barriers, or different career paths	- Quantify applicant and appointment diversity to determine appointment bias at organizational level, and identify specific problem areas; - Ensure that short-lists reflect applicant diversity; - Introduce protocols and training to raise awareness of how to avoid implicit bias in appointment; - Educate staff in recruitment and leadership roles about the benefits of diversity; - Provide alternative entry pathways to attract talent from under-represented groups (with appropriate support).
Departure bias (Eq 4)	If departure bias > 1, efforts to increase diversity will be undermined by lower retention of employees from the under-represented group.	- Quantify and compare diversity in workforce, and in departing employees; - If departure bias exists, investigate and address the causes; - Mentoring and development programs for employees from under-represented groups; - Promote an inclusive work environment and enhance network and sponsoring opportunities (especially for junior colleagues, and colleagues at risk of becoming isolated)

**Fig 5:**
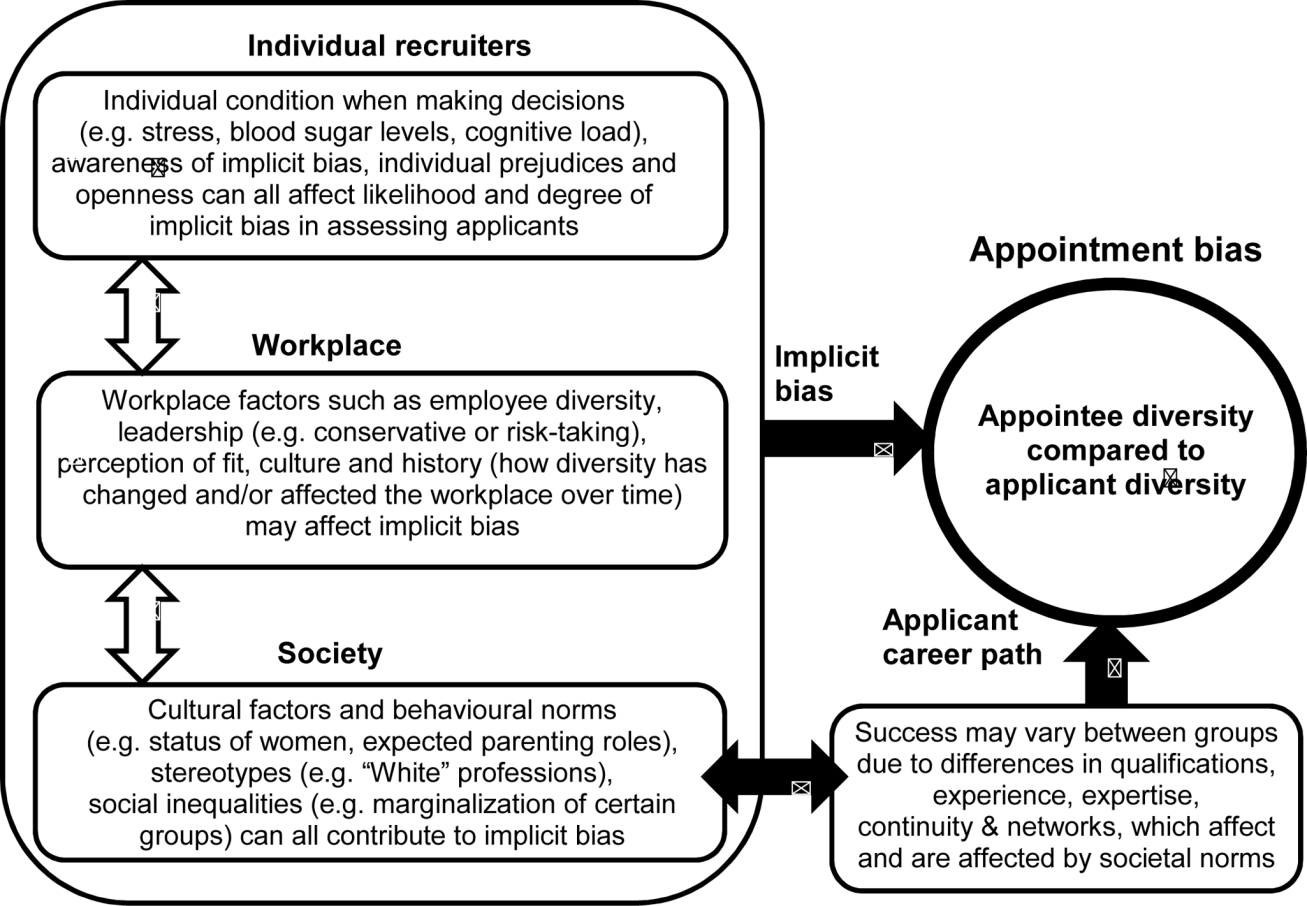
Appointment bias can arise from implicit bias and/or differences in career path.

Where appointment or departure biases exist, or if applicant diversity is low, the specific drivers need to be identified in order to effectively address these barriers to workforce change. However there is unlikely to be a single causal factor for bias or low applicant diversity, because all three measures of inclusion will be affected by multiple factors interacting across a range of scales. For example, preferential appointment of applicants from the dominant group can arise for many reasons (Fig 5). Unconscious or implicit bias favouring “people like us”, and descriptive and prescriptive bias arising from stereotypes is well documented [4,30,50], and can be affected by common stereotypes [51], cultural norms [52], and personal values [53]. Implicit bias is more likely to occur when performance is ambiguous [54] or when people are tired, busy or stressed [55]. Objective decisions are more likely if the potential for implicit bias has been explicitly acknowledged [53,55]. Conversely, when equal opportunities are presumed to exist, implicit bias can become more prevalent when bias is presumed to be absent: this is the “paradox of equality” [56,57].

Hence appointment bias does not arise from a single factor, but from the interaction between processes occurring at individual, workplace and societal scales (Fig 5). Understanding the cause of appointment bias is essential for determining the appropriate response (Table 1). For example, if applicants from under-represented groups have different career paths from other applicants, additional training or alternate recruitment pathways may be required to address appointment bias.

Appointing a diverse group of new employees is only half of the battle: the other challenge is keeping them. In low numbers, people with few role models can feel marginalised and out of place, and struggle to create strong networks and find effective mentors and sponsors [41,47], which can have direct and indirect effects on productivity and success [58]. Stereotypes can also negatively affect how individuals judge the ability of themselves and others [27,59], which can undermine engagement and performance [19,27,60], generating departure bias at multiple points along a career trajectory. Because the individuals affected are rarely able to identfiy the mechanisms at play [4,41], objective evidence such as quantified departure bias from key groups is important for identifing and hence addressing this problem (Table 1).

While we only modelled feedbacks between diversity and bias in appointment and departure, feedbacks also exist between employee and applicant diversity, as depicted in Fig 1. There are a range of factors which discourage people from entering professions and applying to join organisations where they are likely to be in a small minority, and over longer timescales, workforce diversity affects social expectations, role models and stereotypes, affecting inclusion, performance and self-bias, and ultimately contributing to ongoing choices in education and employment which cumulatively affect the diversity of suitably qualified applicants [19,27,52,60–62]. While we did not include these mechanisms in our model, these processes are likely to generate longer-term non-linear feedbacks between diversity within professions, and diversity in recruitment pathways, which may have the potential to create diversity “poverty traps” in different professions.

Addressing applicant diversity in key professions is particularly important for disadvantaged groups. For example, there is a major disparity between the life span and health outcomes of indigenous people and the rest of the Australian population, and increasing the number of indigenous doctors is an important element in resolving this problem [63]. The educational disadvantage which affects many indigenous Australians has been identified as a barrier to entering medical school, and this is being addressed by some Australian universities through premedical preparation courses with flexible entry pathways [17]. While entry requirements may be flexible, exit requirements are not: all graduates must meet the same standards. Hence the diversity of the medical profession is enhanced without compromising academic or professional standards.

The rate at which employees are appointed and depart determines the maximum rate at which workforce diversity can increase, as shown by our simple model. Because only a small proportion of the workforce typically joins or leaves each year, it can take a very long time for employee diversity to change in companies with low turnover rate, even in the absence of any bias towards the current dominant group (Fig 3a). Therefore employee diversity will be disproportionately affected by departure bias during periods of workforce reduction, but periods of expansion provide windows of opportunity for rapid increase diversity.

A recent skills and labour shortage in the Australian mining industry demonstrated how periods of workforce growth provide opportunities to increase diversity. A number of mining companies targeted non-traditional employees to increase the size of the labour pool. For example, hiring new staff for the start-up of two new coal mining operations (Daunia and Caval Ridge) provided BHP Billiton Mitsubishi Alliance (BMA) with an opportunity to rapidly increase workforce diversity. Appointing employees with no mining experience posed a challenge for both recruitment and operations: this was resolved by identifying which roles did not require previous experience in the mining industry (e.g. truck driving), and calculating the numbers of female employees in these roles needed to achieve overall site diversity targets. Interviews for applicants from non-mining backgrounds focussed on cultural fit with the organization and alignment with charter values, e.g. attitudes to safety, work ethic and decision-making [64]. The result is that 23–25% of mine workers at the new Australian BMA mines (Daunia and Caval Ridge) are female, compared to 15% female participation across the sector [65]. Some crews in the truck and shovel fleet have equal numbers of men and women, which is ground-breaking in the coal industry, creating a major shift in workplace culture.

Like all models, our dynamic model of workforce diversity involves numerous simplifications. For example, the model does not account for vertical stratification within an organization, which will affect both appointment and departure bias. Leadership positions are typically occupied by members of the dominant group, who influence appointment, promotion and mentoring, and provide role models for junior employees. Changing diversity at the highest levels of the organization is likely be more difficult than at lower levels in the organisation, due to strong stereotypes about leaders [50], attrition of employees from the non-dominant group at each promotion grade [48], and because people in leadership roles will have larger input to appointment decisions. While our model does not capture the full complexity of individual careers and workforce dynamics, it clearly demonstrate how low diversity can be perpetuated over time by low levels of bias in appointment and departure of individuals from non-dominant groups.

Throughout this paper, we have used the term diversity broadly, encompassing both cognitive (e.g., differences in information perception and processing) and identity (e.g., value and meaning attached to group membership such as gender, ethnicity, education) diversity. Since diversity has many dimensions, it can be difficult to quantify in practice. Furthermore, bringing attention to the group identity of individuals (e.g. singling people out based on gender or ethnicity in discussions of diversity) may be unwelcome and even threatening [40]. Practitioners seeking to apply our results should take care to identify the nature and dimensions of diversity which are important in their specific application, and therefore how to appropriately define, measure and discuss diversity.

## Conclusions

There are numerous imperatives for increasing workforce diversity, and even more reasons why diversity is hard to change. We propose that the key challenge lies in reinforcing feedbacks between group diversity and inclusivity, and our mathematical model demonstrates how these feedbacks can perpetuate a situation of low workforce diversity. Tackling these feedbacks may the key to creating and managing diverse teams.
